# LncRNA *IDH1-AS1* sponges miR-518c-5p to suppress proliferation of epithelial ovarian cancer cell by targeting RMB47

**DOI:** 10.7555/JBR.37.20230097

**Published:** 2023-11-20

**Authors:** Juan Zhou, Yiran Xu, Luyao Wang, Yu Cong, Ke Huang, Xinxing Pan, Guangquan Liu, Wenqu Li, Chenchen Dai, Pengfei Xu, Xuemei Jia

**Affiliations:** 1 Department of Gynecology, Women's Hospital of Nanjing Medical University, Nanjing Maternity and Child Health Care Hospital, Nanjing, Jiangsu 210004, China; 2 Nanjing Maternity and Child Health Medical Institute, Women's Hospital of Nanjing Medical University, Nanjing Maternity and Child Health Care Hospital, Nanjing, Jiangsu 210004, China

**Keywords:** lncRNA, *IDH1-AS1*, epithelial ovarian cancer, miR-518c-5p, RBM47

## Abstract

Long noncoding RNA (lncRNA) *IDH1* antisense RNA 1 (*IDH1-AS1*) is involved in the progression of multiple cancers, but its role in epithelial ovarian cancer (EOC) is unknown. Therefore, we investigated the expression levels of *IDH1-AS1* in EOC cells and normal ovarian epithelial cells by quantitative real-time PCR (qPCR). We first evaluated the effects of *IDH1-AS1* on the proliferation, migration, and invasion of EOC cells through cell counting kit-8, colony formation, EdU, transwell, wound-healing, and xenograft assays. We then explored the downstream targets of *IDH1-AS1* and verified the results by a dual-luciferase reporter, qPCR, rescue experiments, and Western blotting. We found that the expression levels of *IDH1-AS1* were lower in EOC cells than in normal ovarian epithelial cells. High *IDH1-AS1* expression of EOC patients from the Gene Expression Profiling Interactive Analysis database indicated a favorable prognosis, because *IDH1-AS1* inhibited cell proliferation and xenograft tumor growth of EOC. *IDH1-AS1* sponged miR-518c-5p whose overexpression promoted EOC cell proliferation. The miR-518c-5p mimic also reversed the proliferation-inhibiting effect induced by *IDH1-AS1* overexpression. Furthermore, we found that RNA binding motif protein 47 (RBM47) was the downstream target of miR-518c-5p, that upregulation of RBM47 inhibited EOC cell proliferation, and that RBM47 overexpressing plasmid counteracted the proliferation-promoting effect caused by the *IDH1-AS1* knockdown. Taken together, *IDH1-AS1* may suppress EOC cell proliferation and tumor growth *via* the miR-518c-5p/RBM47 axis.

## Introduction

Ovarian cancer is the deadliest cancer of the female reproductive system^[1]^, and epithelial ovarian cancer (EOC) is the most common subtype, accounting for 90% of ovarian cancer cases. EOC has a high incidence with a high mortality rate^[2]^. Although great efforts have been made to treat EOC patients with cytoreductive surgery and chemotherapy as well as targeted therapies, EOC still has high rates of recurrence with a poor prognosis^[3]^. As the pathogenesis of EOC remains unclear, it is urgent to explore the pathogenesis of this disease to identify additional targets for the treatment of EOC.

With the development of human genome sequencing technologies, various nonprotein-coding transcripts have been discovered. Those transcripts with more than 200 nucleotides in length are termed long noncoding RNAs (lncRNAs)^[4]^. It has been widely reported that lncRNAs participate in multiple biological processes, including the regulation of transcriptional and posttranscriptional gene expression. Antisense lncRNAs originating from complementary strands of the protein-coding genes have been reported to participate in the pathogenesis of various diseases, including cancer^[5]^. For example, lncRNA *HIF1A-AS1* induced gemcitabine resistance in patients with pancreatic cancer by upregulating hypoxia-inducible factor-1 alpha (HIF-1α) expression and enhancing glycolysis^[6]^. LncRNA *TRAF3IP2-AS1* suppressed the progression of NONO-TFE3 translocation in renal cell carcinoma by promoting the *PARP1* mRNA decay^[7]^. LncRNA *NR2F1-AS1* regulated NR2F1 translation and ΔNp63 transcription to induce lung metastatic dormancy in breast cancer patients^[8]^.

LncRNA *IDH1* antisense RNA 1 (*IDH1-AS1*) was first discovered to be correlated with the functions of HIF-1α and c-Myc through IDH1 to regulate the Warburg effect, an essential metabolic mechanism for providing energy to maintain the high proliferation of cancer cells^[9]^. *IDH1-AS1* has also been reported to promote cell proliferation and suppress apoptosis of prostate cancer cells *via* autophagy induced by ATG5^[10]^. Additionally, *IDH1-AS1* has been demonstrated to inhibit cell proliferation and tumorigenesis in glioma by altering IDH1 enzymatic activities^[11]^. Because the role of *IDH1-AS1* in EOC has not been reported to date, we conducted a strategy combining bioinformatics analysis and experiments to investigate whether *IDH1-AS1* participates in EOC development, which may assist in identifying new targets for the treatment of EOC.

## Materials and methods

### Cell culture

The human EOC cell lines (3AO, A2780, and OVCAR3) were purchased from Shanghai Mingjin Biotech Co., Ltd. (Shanghai, China). SKOV3, CAOV3, and HEK293T cells were obtained from the Cell Bank of the Chinese Academy of Sciences (Shanghai, China). The normal ovarian epithelial cell line IOSE-386 was provided by Prof. Jin Zhu (General Hospital of Eastern Theater Command, Nanjing, Jiangsu, China). CAOV3, A2780, HEK293T, and IOSE-386 cells were maintained in Dulbecco's modiﬁed Eagle's medium (DMEM; KeyGEN, Jiangsu, China). Specifically, 3AO and OVCAR3 cells were incubated in RPMI-1640 medium (KeyGEN), and SKOV3 cells were cultured in McCoy's 5A medium (KeyGEN). Each culture medium contained 10% fetal bovine serum (FBS; Thermo Fisher Scientiﬁc, Waltham, MA, USA), except for the medium used to culture OVCAR3 cells, which contained 20% FBS.

### Transient transfection

Lipofectamine 3000 transfection reagent (Invitrogen, Carlsbad, CA, USA) was utilized to transfect cells with short interfering RNAs (siRNAs), plasmids, microRNA (miRNA) mimics, or miRNA inhibitors according to the manufacturer's instructions. The siRNAs targeting *IDH1-AS1*, including si-*IDH1-AS1* (1), si-*IDH1-AS1* (2), and si-*IDH1-AS1* (3), as well as negative control siRNA (si-NC), were purchased from RiboBio Co., Ltd. (Cat. Nos. stB0019366A, stB0019366B, stB0019366C, and siN0000001-1-5, Guangzhou, Guangdong, China). The *IDH1-AS1*-overexpressing plasmid (pc-*IDH1-AS1*) and the negative empty vector plasmid (pc-DNA3.1) were constructed by Genecreate Biological Engineering Co., Ltd. (Wuhan, Hubei, China). The miR-518c-5p mimic, miR-518c-5p inhibitor, NC mimic, and NC inhibitor were obtained from RiboBio Co., Ltd. (Cat. Nos. miR10002847, miR20002847, miR1N0000001-1-5, and miR2N000001-1-5).

### Stable transfection

We obtained the *IDH1-AS1*-overexpressing (LV-*IDH1-AS1*) and negative control (LV-NC) recombinant lentiviruses from GenePharma Co., Ltd. (Shanghai, China). SKOV3 cells were seeded into a 24-well plate and incubated overnight, and the cells were transfected, when the cellular confluence reached 30%. The original medium was replaced with 500 μL serum-containing medium with 10% lentivirus fluid, and cells were cultured in the lentiviral solution for 24 h. After the lentivirus-free medium was continuously incubated for 48 h, a concentration of 1 μg/mL puromycin was added to the medium to obtain stably transfected cells.

### RNA extraction and quantitative real-time PCR (qPCR)

Tissue and cell RNAs were extracted according to the instruction of Thermo Scientific GeneJET RNA Purification Kit (Thermo Fisher Scientific), and RevertAid First Strand cDNA Synthesis Kit (Thermo Fisher Scientific) was used to obtain cDNA. The PARIS Kit protein and RNA isolation system (Invitrogen, Carlsbad, CA, USA) was utilized to divide cellular RNA into cytoplasmic and nuclear components. The qPCR assays were performed using SYBR Green dye (Thermo Fisher Scientific) and the following thermocycler program: 5 min at 95 ℃; 15 s at 95 ℃ for 40 cycles; and 1 min at 60 ℃. The relative expression levels of different genes were calculated using the 2^−ΔΔCT^ method. *ACTB*, *GAPDH*, and *U6* were utilized as internal controls. The primer sequences are listed in ***Table 1***. miRNA-specific stem-loop *U6* primers were constructed from RiboBio Co., Ltd. (Cat. #MQPS0000002-1-100).

**Table 1:** Primer sequences for quantitative real-time PCR

Genes	Forward (5′-3′)	Reverse (5′-3′)
*IDH1-AS1*	GTCATGGAGGTGTCTGTGTTAG	GTCACTCTGCGGATGTTTCT
*RBM47*	TGATGGACTTTGACGGCAAGA	GGGCGGATCTCGTAGTTGTT
*ZNF621*	CTCCAAACAACTTGGCCTCAG	GAATGGAAACGCTACCAGAGAA
*CASTOR2*	GCCACCACCCTCATAGATGT	AGGAGGTCACTGGGGAACTT
*U6*	CTCGCTTCGGCAGCACA	AACGCTTCACGAATTTGCGT
*ACTB*	TCCCTGGAGAAGAGCTACGA	AGCACTGTGTTGGCGTACAG
*GAPDH*	CAATGACCCCTTCATTGACC	TTGATTTTGGAGGGATCTCG

### Cell counting kit-8 (CCK-8) assay

After cell transfection for 24 h, approximately 2000 to 3000 cells in 100 μL of medium were added to each well of a 96-well plate. After allowing the cells to adhere for 4 to 6 h, 10 µL of CCK-8 solution (KeyGEN) was added to each well of the plate followed by incubation for 2 h. The absorbance was read at 450 nm using a BioTek Synergy H4 (BioTek, Winooski, Vermont, USA), which was considered the 0-h time point. The same process was performed on additional cells at the same time in the next three days, representing the 24-h, 48-h, and 72-h time points.

### Colony formation assay

SKOV3 and A2780 cells were seeded and transfected with siRNAs, overexpression plasmid, or miRNA mimics in six-well plates. After the cells were digested and counted, 2000 to 3000 cells were re-cultured in new six-well plates. The cells were allowed to adhere and grow for 7 days to 2 weeks. Cells were then fixed with 1 mL of paraformaldehyde for 30 to 60 min and stained with crystal violet solution for approximately 30 min. The number of colonies was photographed and counted using ImageJ software (National Institutes of Health, Bethesda, MD, USA).

### EdU assay

SKOV3 cells (1.5 × 10^4^) or A2780 cells (3 × 10^4^) were seeded in each well of a 96-well plate and cultured for 24 h. Cell-Light EdU DNA Apollo In Vitro Kit (RiboBio) was applied to perform the EdU assay. The cells were labeled with 50 mmol/L EdU solution for 2 h, and stained with Apollo and Hoechst 33342 Dye Solution (RiboBio) in sequence after been fixed with 4% paraformaldehyde. Axio Observer D1 fluorescence microscope (Carl Zeiss, Jena, Germany) was used to take photographs of the stained cells.

### Transwell assay

Medium (600 μL) containing 20% FBS was added to the lower chamber of the Transwell (Corning Inc., Corning, NY, USA), and 200 μL of serum-free medium was added to the upper chamber. SKOV3 cells (3 × 10^4^) or A2780 cells (5 × 10^4^) were seeded into Transwell chambers without or with Matrigel (BD Biosciences, San Jose, CA, USA) (60 μL/well; medium/Matrigel ratio, 7∶1) to conduct migration or invasion assays, respectively. Following 24 to 72 h of cultivation, the chamber was fixed with 4% paraformaldehyde (BioSharp, Hefei, Anhui, China) and then stained with 0.1% crystal violet solution (BioSharp). Cells were wiped away from the upper surface of the chamber using a cotton swab, and images of the lower surface of the chamber were acquired for randomly selected areas using an Axio Observer D1 fluorescence microscope (Carl Zeiss).

### Wound-healing assay

SKOV3 and A2780 cells were cultured in six-well plates with medium containing 10% FBS. When the cellular confluence reached more than 95%, horizontal lines were made on the backside of the plate, and longitudinal lines were scratched in the cell bed using 200 μL pipette tips. The FBS-containing medium was then replaced with a serum-free medium. Images were acquired at 0, 24, and 48 h after the wound was generated using an EVOS XL Core digital microscope (Thermo Fisher Scientific) and analyzed by ImageJ software.

### Xenografts in nude mice

Ten female BALB/c nude mice aged 6 weeks were purchased from the Model Animal Research Center of Nanjing University (Nanjing, Jiangsu, China). The nude mice were kept in a specific pathogen-free environment and randomly and equally divided into the LV-*IDH1-AS1* group and the LV-NC group (*n* = 5 for each group). SKOV3 cells (9 × 10^6^) transfected with LV-*IDH1-AS1* or LV-NC in 200 μL of PBS solution were subcutaneously injected into the left axilla of the LV-*IDH1-AS1* group or the LV-NC group nude mice, respectively. The tumor size was measured every 2 to 3 days using manual calipers, and the tumor volume was calculated according to the following formula: Tumor volume = 0.5 × length × width^2^. The tumors were resected 25 days after injection of cells. All animal protocols were approved by the Animal Ethical and Welfare Committee of Nanjing Medical University (Approval No. IACUC-1911022).

### Dual-luciferase reporter assay

Bioinformatics tools were utilized to predict the binding site between *IDH1-AS1* and miR-518c-5p. The wild type (WT) and mutant type (MT) plasmids of *IDH1-AS1* were inserted into the pmirGLO plasmid. A total of 250 ng of pmirGLO-*IDH1-AS1*-WT or pmirGLO-*IDH1-AS1*-MT and 250 ng of miR-518c-5p mimics or NC mimics were cotransfected into HEK293T cells with 2.5 μL of Lipofectamine 3000 and 0.5 μL of P3000 (Invitrogen) for 48 h. Luciferase activities of Firefly and Renilla were measured using the Dual-Luciferase Reporter Gene Assay Kit (Promega, Madison, WI, USA) and detected by the GloMax 96-well fluorescence detector (Promega) according to the manufacturer's instructions. The ratio of firefly to Renilla luciferase activity was calculated as the relative luciferase activity. To verify the targeting relevance between miR-518c-5p and the 3′ UTR of *RBM47*, the same procedure was used but with different plasmids, respectively.

### Western blotting analysis

Total protein was isolated by lysing cells with RIPA lysis buffer (Servicebio, Wuhan, Hubei, China), and the protein concentration was measured using a BCA Protein Assay Kit (Beyotime, Shanghai, China). Protein samples were separated by SDS-PAGE and transferred onto PVDF membranes (Millipore, Boston, MA, USA). After blocking the membranes with 5% skim milk, the membranes were incubated with the following primary antibodies at 4 ℃ overnight: anti-β-actin antibody (1∶2000 dilution; Cat. No. GB15003, Servicebio) and anti-RBM47 antibody (1∶5000 dilution; Cat. No. ab167164, Abcam, Cambridge, UK). The membranes were then incubated with the secondary antibody for 2 h at 37 ℃. Protein bands were visualized by using the FluuroChem M imaging system (Protein Simple Inc., San Jose, CA, USA) according to the manufacturer's instructions.

### Immunohistochemistry

Tissue specimens were paraffin-embedded and sliced into sections that were dewaxed with xylene and dehydrated with gradient alcohol. The slices were heated in citrate buffer, followed by blocking with 3% H_2_O_2_, and sealed with goat serum. The sections were incubated with primary antibody overnight at 4 ℃, including anti-RBM47 antibody (1∶100 dilution; Cat. No. ab167164, Abcam), anti-Ki67 antibody (1∶300 dilution; Cat. No. NB500-170, Novus Biologicals, Littleton, CO, USA), and then incubated with HRP-labeled secondary antibody (1∶500 dilution; Cat. No. abs20040ss, Absin Bioscience, Shanghai, China). The slices were treated with 3,39-diaminobenzidine tetrahydrochloride reagent to develop color, and re-stained with hematoxylin. Each section was photographed under the microscope (Carl Zeiss) at 400× magnification. Integrated optical density summation of RRBM47 and Ki67 protein expression was calculated by Image-Pro Plus software (Media Cybernetics, Silver Spring, MD, USA).

### Bioinformatics analysis

The association between *IDH1-AS1* and the prognosis of EOC patients was analyzed using the GEPIA database (http://gepia.cancer-pku.cn). The clinical data of EOC patients were obtained from The Cancer Genome Atlas (TCGA) database (http://cancergenome.nih.gov), and the *IDH1-AS1* expression data of EOC patients were acquired from the Atlas of non-coding RNA in Cancer (TANRIC) database (http://ibl.mdanderson.org/tanric/_design/basic/index.html). The downstream miRNA targets of *IDH1-AS1* were searched from the RNA22 (https://cm.jefferson.edu/rna22/), miRDB (http://www.mirdb.org/), and LncRNASNP2 (http://bioinfo.life.hust.edu.cn/lncRNASNP/) databases. The downstream gene targets of miR-518c-5p were screened from the miRWalk (http://mirwalk.umm.uni-heidelberg.de/) database. RNAHybrid (v2.2) software (https://bibiserv.cebitec.uni-bielefeld.de/rnahybrid/) was used to predict binding sites between *IDH1-AS1* and miR-518c-5p as well as between miR-518c-5p and the 3′ UTR of *RBM47*.

### Statistical analysis

The data of cellular experiments were presented as the mean ± standard deviation of three independent experiments, and the data of animal tests were presented as mean ± standard error of the mean. The Chi-square (*χ*^2^) test was applied to compare the rates (%) of the two groups. The Cox proportional hazards regression model was used to analyze the association between clinical data and survival of EOC patients. Two-tailed Student's *t-*test and two-way analysis of variance (ANOVA) followed by Dunnett's tests were applied to compare the data between two or more groups by using GraphPad Prism 7 (GraphPad Software, La Jolla, CA, USA). *P* < 0.05 was considered statistically significant.

## Results

### *IDH1-AS1* was decreased in EOC cells and associated with the prognosis of EOC

**Figure 1:**
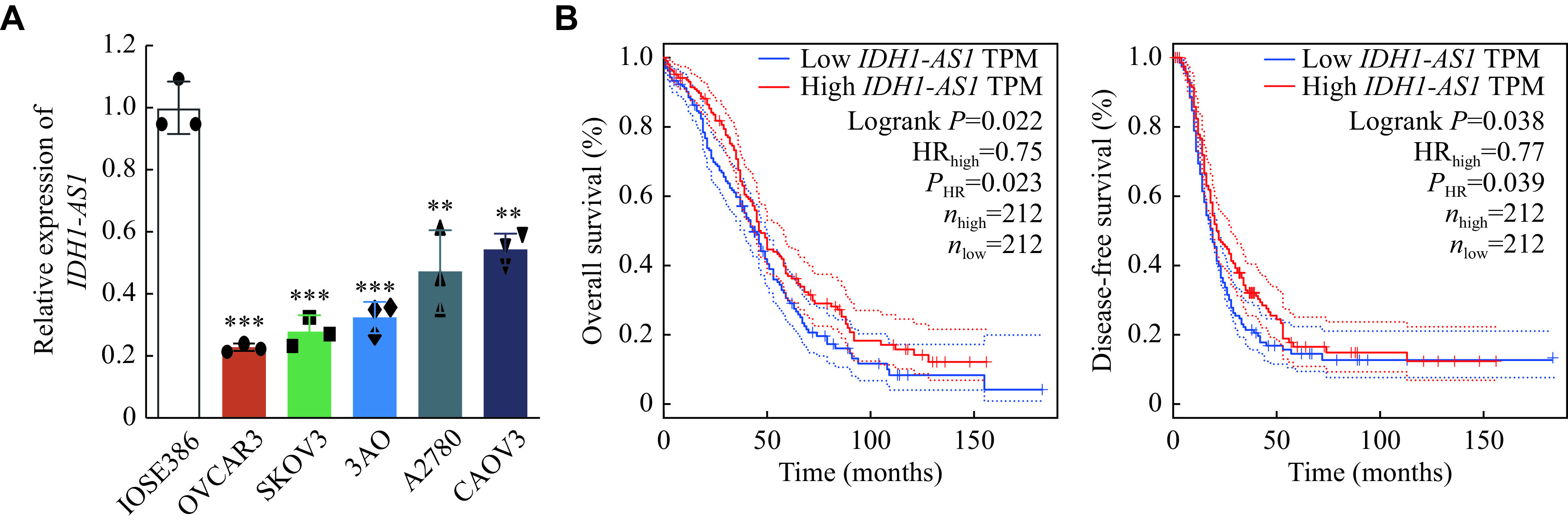
*IDH1-AS1* was downregulated in EOC cells and was associated with the prognosis of EOC.

To identify the potential effects of *IDH1-AS1* on EOC, we first investigated the expression levels of *IDH1-AS1* in EOC cell lines. The abundance of *IDH1-AS1* was lower in EOC cells (OVCAR3, SKOV3, 3AO, A2780, and CAOV3) than in normal ovarian epithelial cells (IOSE386) (***Fig. 1A***). Because SKOV3 and A2780 cells were the commonly used EOC cells, we selected them for further investigation. We next detected *IDH1-AS1* expression in EOC cells from the patients included in the GEPIA, TCGA, and TANRIC databases. High *IDH1-AS1* expression was associated with an increased overall survival (OS) and disease-free survival in EOC patients according to the GEPIA database (***Fig. 1B***). Furthermore, we downloaded clinical information of EOC patients from TCGA and the expression data of *IDH1-AS1* from the TANRIC database. Patients with incomplete primary clinical data or lacking *IDH1-AS1* expression data were excluded. A total of 410 EOC patients were included in this analysis. The *IDH1-AS1* expression levels were not associated with the Federation International of Gynecology and Obstetrics (FIGO) stage, grade, lymph node invasion or neoplasm status of EOC, but with age and race (***Table 2***). According to univariate and multivariate Cox regression analyses, *IDH1-AS1* expression level may be an independent predictor for OS of EOC patients (***Tables 3*** and ***4***). Taken together, these results indicated that the expression levels of *IDH1-AS1* were decreased in EOC cells and that low *IDH1-AS1* expression was associated with a poor prognosis in EOC patients.

**Table 2:** Correlation between *IDH1-AS1* expression and clinicopathological features in patients with epithelial ovarian cancer

Clinicopathological features	Cases^a^ (*n*)	IDH1-AS1 expression		*P*-value^b^
		Low^c^	High^c^	
Age (years)	409			0.042^*^
<60	216	98 (45.4)	118 (54.6)	
≥60	193	107 (55.4)	86 (44.6)	
Race	397			0.022^*^
White	360	188 (52.2)	172 (47.8)	
Others	37	12 (33.4)	25 (66.6)	
Neoplasm status^d^	358			0.395
With tumor	266	138 (51.9)	128 (48.1)	
Tumor free	92	43 (46.7)	49 (53.3)	
Site	384			0.726
Bilateral	285	141 (49.5)	144 (50.5)	
Unilateral	99	51 (51.5)	48 (48.5)	
Stage^e^	407			0.392
Ⅱ	21	11 (52.4)	10 (47.6)	
Ⅲ	325	156 (48.0)	169 (52.0)	
Ⅳ	61	35 (57.4)	26 (42.6)	
Grade	410			0.668
G1/G2/GX^f^	57	30 (52.6)	27 (47.4)	
G3/G4	353	175 (49.6)	178 (50.4)	
Invasion	158			0.314
Yes	104	55 (52.9)	49 (47.1)	
No	54	24 (44.4)	30 (55.6)	
Overall status	410			0.221
Deceased	256	134 (52.3)	122 (47.7)	
Alive	154	71 (46.1)	83 (53.9)	
^a^The data for "cases" in the second column represent the actual patient cases for statistics, which might be less than the total patient cases because of the missing data in the database. ^b^*P-*values were determined by using the Chi-square test. ^*^P < 0.05. ^c^The low expression group and high expression group were defined according to the median of *IDH1-AS1* level.^d^Neoplasm status indicates the state or condition of an individual's tumor at a specific time, including with tumor and tumor free. ''Tumor free'' means the tumor has been removed while ''With tumor'' refers to the presence of a tumor.^e^Stage was classified according to the Federation International of Gynecology and Obstetrics (FIGO) criterion.^f^GX indicates the undefined grade. The case number of G1, G2, G3, G4 and GX were 1, 47, 352, 1, and 9, respectively.				

**Table 3:** Univariate and multivariate Cox regression analyses of overall survival in patients with epithelial ovarian cancer

Variables^a^	Overall survival						
	Univariate analysis				Multivariate analysis		
	*n*	HR (95% CI)	*P*		*n*	HR (95% CI)	*P*
Age (years, <60 *vs.* ≥60)	409	1.021 (1.010–1.033)	<0.001^*^		409	1.017 (1.005–1.030)	0.007^*^
Race (white *vs.* others)	397	1.626 (1.004–2.635)	0.048^*^		397	1.514 (0.916–2.504)	0.106
Neoplasm status^b^ (with tumor *vs.* tumor free)	358	0.123 (0.070–0.216)	<0.001^*^		358	0.129 (0.073–0.227)	<0.001^*^
Site (bilateral *vs.* unilateral)	384	1.337 (0.897–1.993)	0.154				
Stage^c^ (Ⅱ *vs.* Ⅲ *&* Ⅳ)	407	1.276 (0.971–1.677)	0.080				
Grade (G1/G2/GX *vs.* G3/G4)	410	1.109 (0.781–1.574)	0.562				
Invasion (yes *vs.* no)	158	0.855 (0.528–1.385)	0.525				
*IDH1-AS1* expression (low *vs.* high)	410	0.729 (0.570–0.932)	0.012^*^		410	0.727 (0.555–0.952)	0.020^*^
^a^The first variable was the reference variable in each group. ^b^Neoplasm Status indicates the state or condition of an individual's tumor at a specific time, including with tumor and tumor free. ''Tumor free'' means the tumor has been removed while ''With tumor'' refers to the presence of a tumor.^c^Stage was classified according to the Federation International of Gynecology and Obstetrics (FIGO) criterion.*P*-values were determined by univariate and multivariate Cox regression analyses. When the factors with a *P* < 0.05 in the univariate analyses, they were subjected to multivariate analysis. ^*^P < 0.05. Abbreviation: HR, hazard ratio; CI, confidence interval; GX, undefined grade.							

**Table 4:** Univariate and multivariate Cox regression analyses of disease-free survival in patients with epithelial ovarian cancer

Variables^ a^	Disease-free survival						
	Univariate analysis				Multivariate analysis		
	*n*	HR (95% CI)	*P*		*n*	HR (95% CI)	*P*
Age (years, <60 *vs.* ≥60)	409	1.008 (0.996–1.019)	0.189				
Race (white *vs.* others)	397	1.291 (0.831–2.006)	0.255				
Neoplasm status^b^ (with tumor *vs.* tumor free)	358	0.161 (0.107–0.244)	<0.001^*^		358	0.165 (0.109–0.250)	<0.001^*^
Site (bilateral *vs.* unilateral)	384	0.804 (0.597–1.083)	0.151				
Stage^c^ (Ⅱ *vs.* Ⅲ *&.* Ⅳ)	407	1.309 (1.001–1.711)	0.049^*^		407	0.794 (0.617–1.022)	0.073
Grade (G1/G2/GX *vs.* G3/G4)	410	1.156 (0.801–1.668)	0.438				
Invasion (yes *vs.* no)	158	1.019 (0.645–1.610)	0.934				
*IDH1-AS1* expression (low *vs.* high)	410	0.812 (0.635–1.037)	0.095				
^a^The first variable was the reference variable in each group. ^b^Neoplasm Status indicates the state or condition of an individual's tumor at a specific time, including with tumor and tumor free. ''Tumor free'' means the tumor has been removed while ''With tumor'' refers to the presence of a tumor.^c^Stage was classified according to the Federation International of Gynecology and Obstetrics (FIGO) criterion. *P*-values were determined by univariate and multivariate Cox regression analyses. When the factors with a *P* < 0.05 in the univariate analyses, they were subjected to multivariate analysis. ^*^P < 0.05. Abbreviations: HR, hazard ratio; CI, confidence interval; GX, undefined grade.							

### *IDH1-AS1* inhibited the proliferation of EOC cells *in vitro*

**Figure 2:**
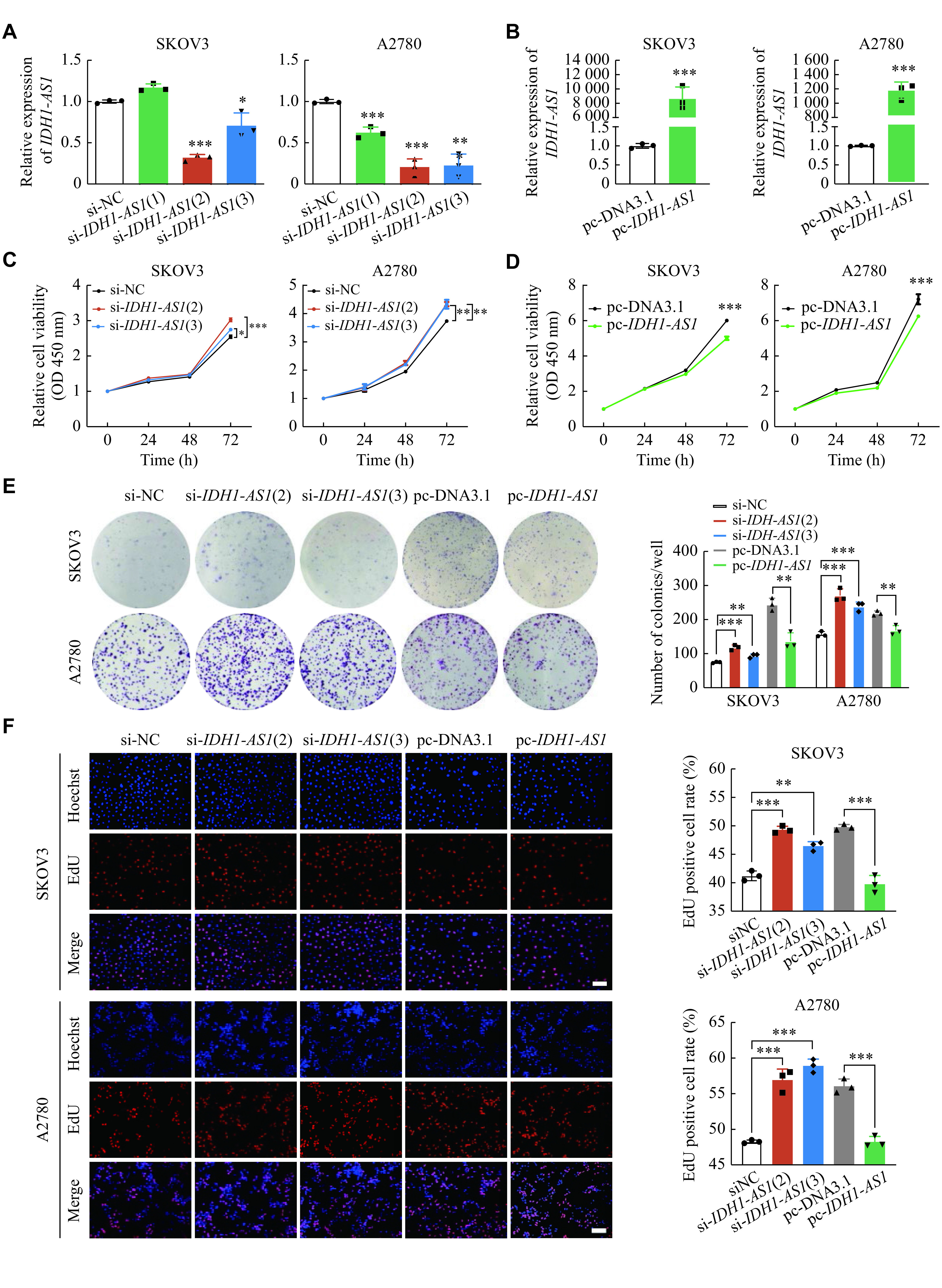
*IDH1-AS1* inhibited the proliferation of EOC cells *in vitro.*

Given the effect of *IDH1-AS1* on the prognosis of EOC, we further investigated the influence of *IDH1-AS1* on cellular proliferation, migration, and invasion by knocking down or overexpressing *IDH1-AS1* in EOC cells with siRNAs or overexpressing plasmid, respectively (***Fig. 2A*** and ***2B***). The CCK-8 assay results demonstrated that the downregulation of *IDH1-AS1* promoted EOC cell viability, while the overexpression of *IDH1-AS1* significantly suppressed EOC cell viability (***Fig. 2C*** and ***2D***). Moreover, both the colony formation and the EdU assays were utilized to evaluate cell proliferation, and the trends were similar to those in the CCK-8 assays (***Fig. 2E*** and ***2F***). Transwell assays and wound-healing assays indicated that *IDH1-AS1* had no significant effect on the invasion and migration of EOC cells (***Supplementary Fig. 1A*** and ***1B***, available online). Together, these results indicated that *IDH1-AS1* inhibited EOC cell proliferation *in vitro*.

### *IDH1-AS1* acted by sponging miR-518c-5p in EOC cells

**Figure 3:**
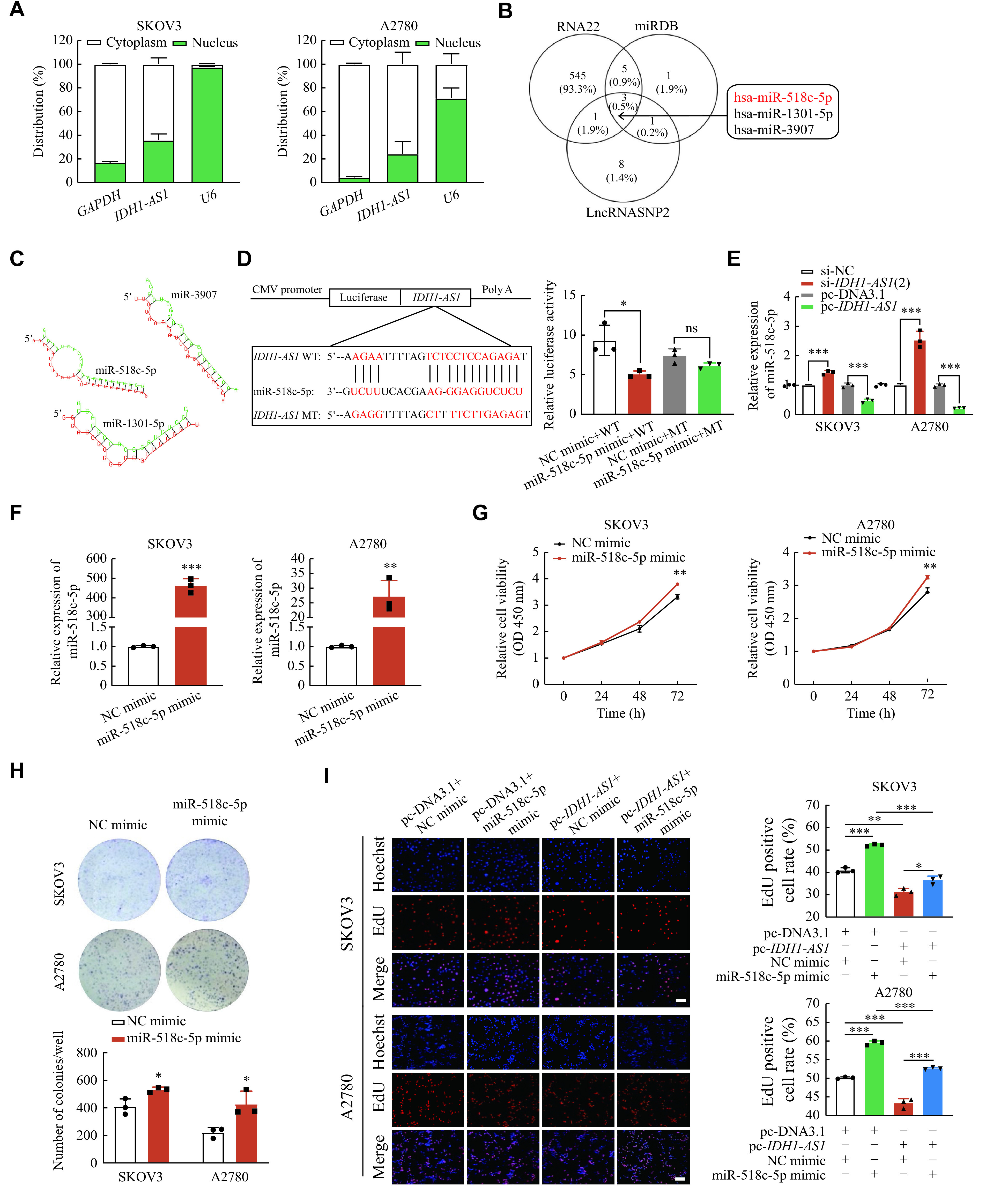
*IDH1-AS1* acted by sponging miR-518c-5p in EOC cells.

To investigate the mechanism of *IDH1-AS1* in EOC cells, the nuclear and cytoplasmic RNA isolation assay was applied to define its cellular location. *IDH1-AS1* was confirmed to be distributed mainly in the cytoplasm of EOC cells (***Fig. 3A***). The databases of RNA22, miRDB, and LncRNASNP2 were then utilized to screen downstream miRNA targets, which identified miR-518c-5p, miR-1301-5p, and miR-3907 (***Fig. 3B***). The number of consecutive binding sites between *IDH1-AS1* and miR-518c-5p was predicted to be the largest among these miRNAs by RNAhybrid software (***Fig. 3C***), which indicated their greatest binding possibility. Dual-luciferase reporter analysis and qPCR assays were performed to verify the correlation between *IDH1-AS1* and miR-518c-5p. The results showed that miR-518c-5p upregulation suppressed the luciferase activity of *IDH1-AS1*-WT but did not affect the luciferase activity of *IDH1-AS1*-MT (***Fig. 3D***). The expression level of miR-518c-5p was increased after *IDH1-AS1* knockdown but decreased after *IDH1-AS1* overexpression in EOC cells (***Fig. 3E***). Subsequently, transfection of miR-518c-5p mimic into SKOV3 and A2780 cells (***Fig. 3F***) promoted cell viability and colony formation in EOC cells (***Fig. 3G*** and ***3H***). Rescue experiments of the EdU assay further indicated that the miR-518c-5p mimic reversed the proliferation inhibiting effect induced by overexpression of *IDH1-AS1* in EOC cells (***Fig. 3I***). Thus, these findings suggest that *IDH1-AS1* may serve as a suppressor by sponging miR-518c-5p in EOC cells.

### *IDH1-AS1* affected EOC cells by targeting the miR-518c-5p/RBM47 axis

**Figure 4:**
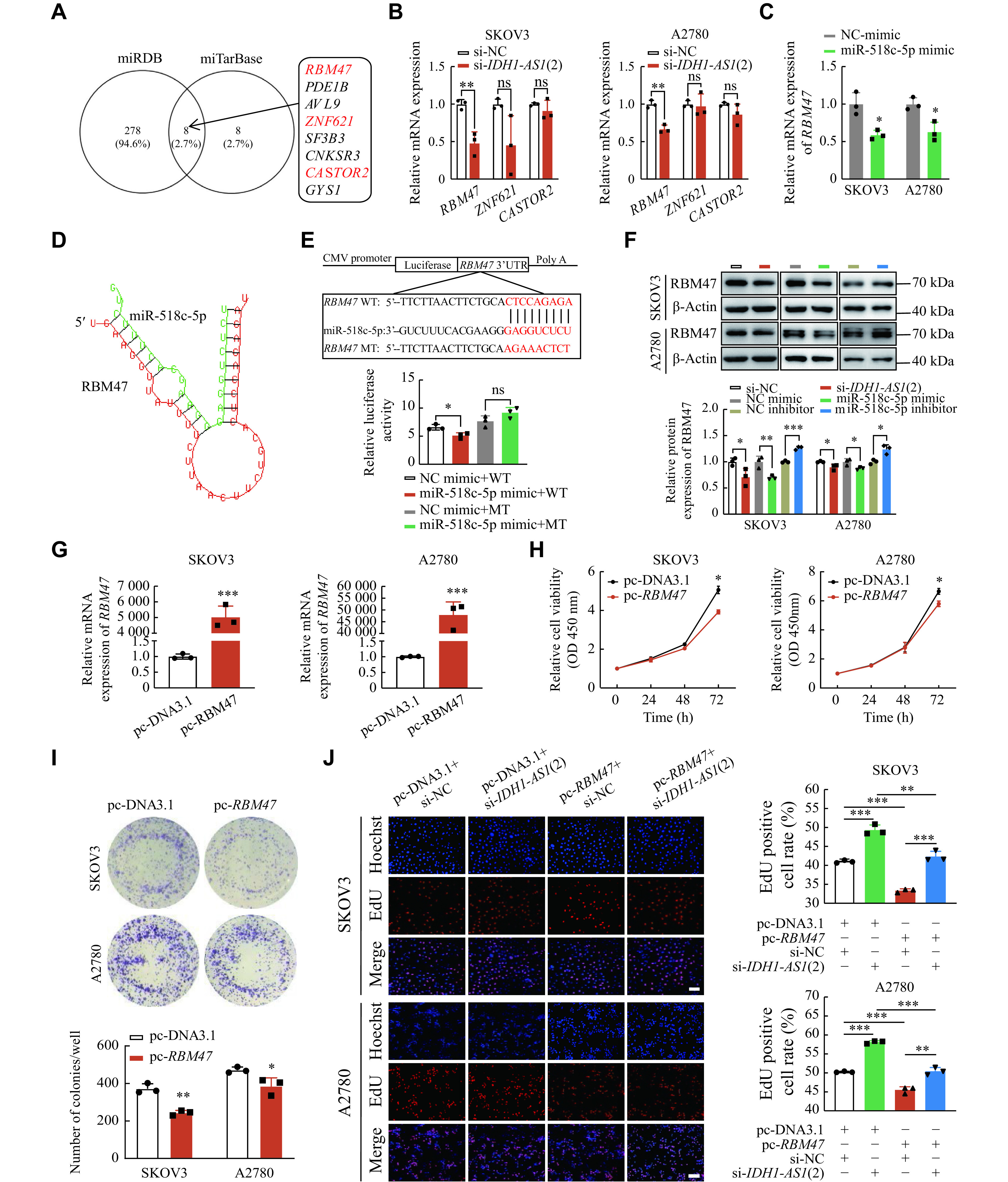
*IDH1-AS1* affected EOC cells by targeting the miR-518c-5p/RBM47 axis.

To identify the putative targets of miR-518c-5p, we used the miRWalk website, including the miRDB and miTarBase databases, and identified eight shared genes (***Fig. 4A***). Because previous studies have reported that RNA binding motif protein 47 (RBM47), zinc finger protein 621 (ZNF621), and cytosolic arginine sensor for mTORC1 subunit 2 (CASTOR2) function as tumor suppressors, we selected these genes for further verification. The qPCR analysis showed that only the mRNA level of *RBM47* was significantly decreased after *IDH1-AS1* knockdown in EOC cells (***Fig. 4B***). The mRNA expression of *RBM47* was also inhibited after miR-518c-5p overexpression (***Fig. 4C***). Next, we predicted the binding sites between 3′ UTR of *RBM47* and miR-518c-5p by the RNAhybrid software (***Fig. 4D***). A dual-luciferase reporter assay revealed that the luciferase activity of *RBM47*-WT was significantly decreased when cotransfected with the miR-518c-5p mimic, but this change did not occur in the luciferase activity of *RBM47*-MT (***Fig. 4E***). Furthermore, Western blotting analysis indicated the protein level of RBM47 was decreased after *IDH1-AS1* suppression or miR-518c-5p overexpression, but increased after miR-518c-5p suppression in EOC cells (***Fig. 4F***). Furthermore, *RBM47* overexpression (***Fig. 4G***) inhibited cell viability and colony formation in EOC cells (***Fig. 4H*** and ***4I***). Rescue experiments of the EdU assay revealed that the *RBM47* overexpressing plasmid counteracted the proliferation-promoting effect caused by the *IDH1-AS1* knockdown in EOC cells (***Fig. 4J***). Taken together, these results demonstrate that *IDH1-AS1* may inhibit the proliferation of EOC cells *via* the miR-518c-5p/RBM47 axis.

### *IDH1-AS1* suppressed tumor growth of EOC cells *in vivo*

**Figure 5:**
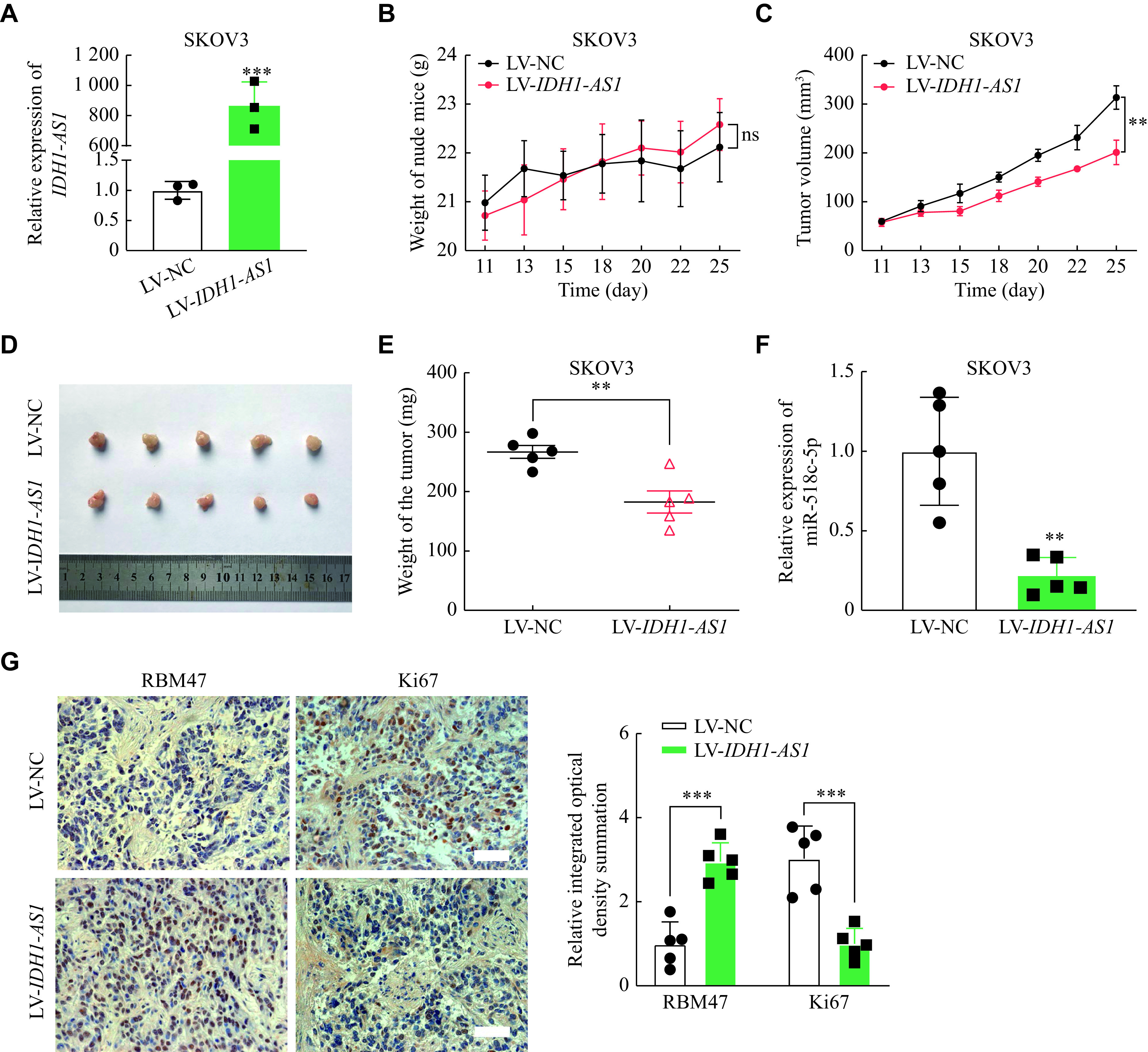
*IDH1-AS1* suppressed tumor growth of EOC *in vivo.*

Considering the suppressive effects of *IDH1-AS1* on EOC *in vitro*, we further investigated its function *in vivo*. The qPCR confirmed that the expression of *IDH1-AS1* was significantly higher in SKOV3 cells transfected with LV-*IDH1-AS1*, compared with the control cells (***Fig. 5A***). There was no significant difference in the weight of nude mice after SKOV3 cell injection in the LV-NC group and LV-*IDH1-AS1* group (***Fig. 5B***). The tumor growth curve showed that the tumor volume of nude mice was significantly lower in the LV-*IDH1-AS1* group than in the control group (***Fig. 5C***). Subcutaneous tumors were dissected from nude mice after cells injection for 25 days (***Fig. 5D***), and the tumor weight was significantly lower in the LV-*IDH1-AS1* group than in the control group (***Fig. 5E***). The qPCR also showed that the expression of miR-518c-5p was obviously lower in subcutaneous tumors of the LV-*IDH1-AS1* group (***Fig. 5F***). Then, immunohistochemistry was performed to detect RBM47 and Ki67 protein expression in tumor tissues, identifying that RBM47 protein was highly expressed, while Ki67 protein was lowly expressed in *IDH1-AS1*-overexpressing tumor tissues (***Fig. 5G***). These results suggested that *IDH1-AS1* suppressed xenograft tumor growth of EOC cells *in vivo*.

## Discussion

Accumulating evidence indicates that lncRNAs participate in the progression of EOC^[12]^. *IDH1-AS1* is an antisense lncRNA derived from the complementary strand of the *IDH1* gene. *IDH1-AS1* has recently been demonstrated to affect cell proliferation and tumorigenesis of cervical cancer, prostate cancer, and glioblastoma^[9–11]^, but it remains unknown whether *IDH1-AS1* plays a role in the pathogenesis of EOC cells.

In the present study, we identified that low expression of *IDH1-AS1* was an independent predictor for a shorter OS in EOC patients based on the analyses using TCGA and TANRIC data. Moreover, an increasing age was associated with an increased risk of EOC progression, which was consistent with the previous reports^[13–14]^. The present results also indicated that the FIGO stage, grade, and lymph node invasion were not indicators of OS and disease-free survival in EOC. Although the grade was considered a controversial prognostic marker^[13–14]^, the FIGO stage and lymph node invasion have been indicated as significant prognostic factors of EOC^[15–18]^. These differences may be due to the fact that the clinical information provided by the databases used in the present study was only from serous EOC patients, and the clinical data were incomplete, especially the data on lymph node metastasis. Therefore, it is necessary to perform further analysis to precisely clarify the association between *IDH1-AS1* and clinical features.

Similar to the study conducted by Xiang *et al*^[9]^ in HeLa cells, we demonstrated that silencing *IDH1-AS1* enhanced EOC cell proliferation, while overexpressing *IDH1-AS1* attenuated EOC cell growth. Wang J *et al*^[11]^ also reported that *IDH1-AS1* suppressed glioblastoma cell proliferation and tumorigenesis. However, Zhang *et al*^[10]^ revealed that *IDH1-AS1* knockdown promoted cell apoptosis in addition to inhibiting cell proliferation and tumor growth in prostate cancer. These studies indicate that different effects of *IDH1-AS1* may result from different mechanisms in various cancers.

*IDH1-AS1* has been demonstrated to participate in the progression of multiple cancers by regulating the IDH1 activity^[9,11,19]^. In the present study, we found that *IDH1-AS1* was mainly distributed in the cytoplasm of EOC cells. It is possible that *IDH1-AS1* functions in EOC through some other pathways, and some evidence suggests that different subcellular localizations may present distinct biological functions of lncRNAs^[20–21]^. The ceRNA hypothesis is the widely accepted mechanism for cytoplasmic lncRNAs that are suggested to compete against protein-coding mRNAs for the miRNA binding^[12]^. Currently, lncRNA-miRNA-mRNA interactions attract much attention, because they play an extensive role in the pathogenesis of EOC^[22–24]^. In the present study, we demonstrated that *IDH1-AS1* suppressed EOC cell proliferation and tumor growth by sponging miR-518c-5p.

miR-518c-5p is a member of the miR-515 family that belongs to the largest human miRNA cluster on chromosome 19, known as C19MC, encoding as many as 59 mature miRNAs^[25]^. miR-518c-5p has been reported to be involved in the progression of bladder cancer in a multivariate Cox regression analysis^[26]^. A reported miRNA microarray analysis has indicated that miR-518c-5p is also upregulated in retinoblastoma and related to tumorigenesis^[27]^. In addition, miR-518c-5p has been suggested to participate in the growth and metastasis of oral cancer by targeting the SDF-1/CXCR4 system^[28]^. In line with these findings, miR-518c-5p may promote EOC cell proliferation in the present study as an oncomiR^[25]^. However, another study has reported that hsa_circ_0007843 promotes the progression of colon cancer by sponging miR-518c-5p to regulate MMP2 expression^[29]^. As the above-mentioned functions of miR-518c-5p range from oncomiR to tumor suppressor, our findings have suggested that the effect of miR-518c-5p is determined by the cellular context in which it is activated.

As an RNA-binding motif protein, RBM47 is a conserved RNA-binding protein in vertebrates and proved to be essential for the viability and growth of vertebrate embryo^[30]^. RBM47 significantly regulates mRNA transcription, RNA splicing, and RNA transportation^[31]^. It is widely accepted that RBM47 functions as a tumor suppressor in multiple cancers. Sakurai T *et al*^[32]^ demonstrated that RBM47 suppressed tumor growth of lung adenocarcinoma by inhibiting Nrf2 function. Guo *et al*^[33]^ reported that RBM47 acted as a DNA/RNA modulator to inhibit the progression of hepatocellular carcinoma. Qin *et al*^[34]^ found that RBM47 suppressed cell proliferation and activated autophagy in papillary thyroid carcinoma. Shen *et al*^[35]^ revealed that RBM47 stabilized *AXIN1* mRNA to suppress Wnt/β-catenin signaling and inhibited tumor proliferation and metastasis in non-small cell lung cancer. Consistent with these investigations, the present study showed that RBM47 suppressed EOC cell proliferation, and thus we concluded that *IDH1-AS1* inhibited EOC cell proliferation and tumor growth by upregulating RBM47 mediated by miR-518c-5p.

**Figure 6:**
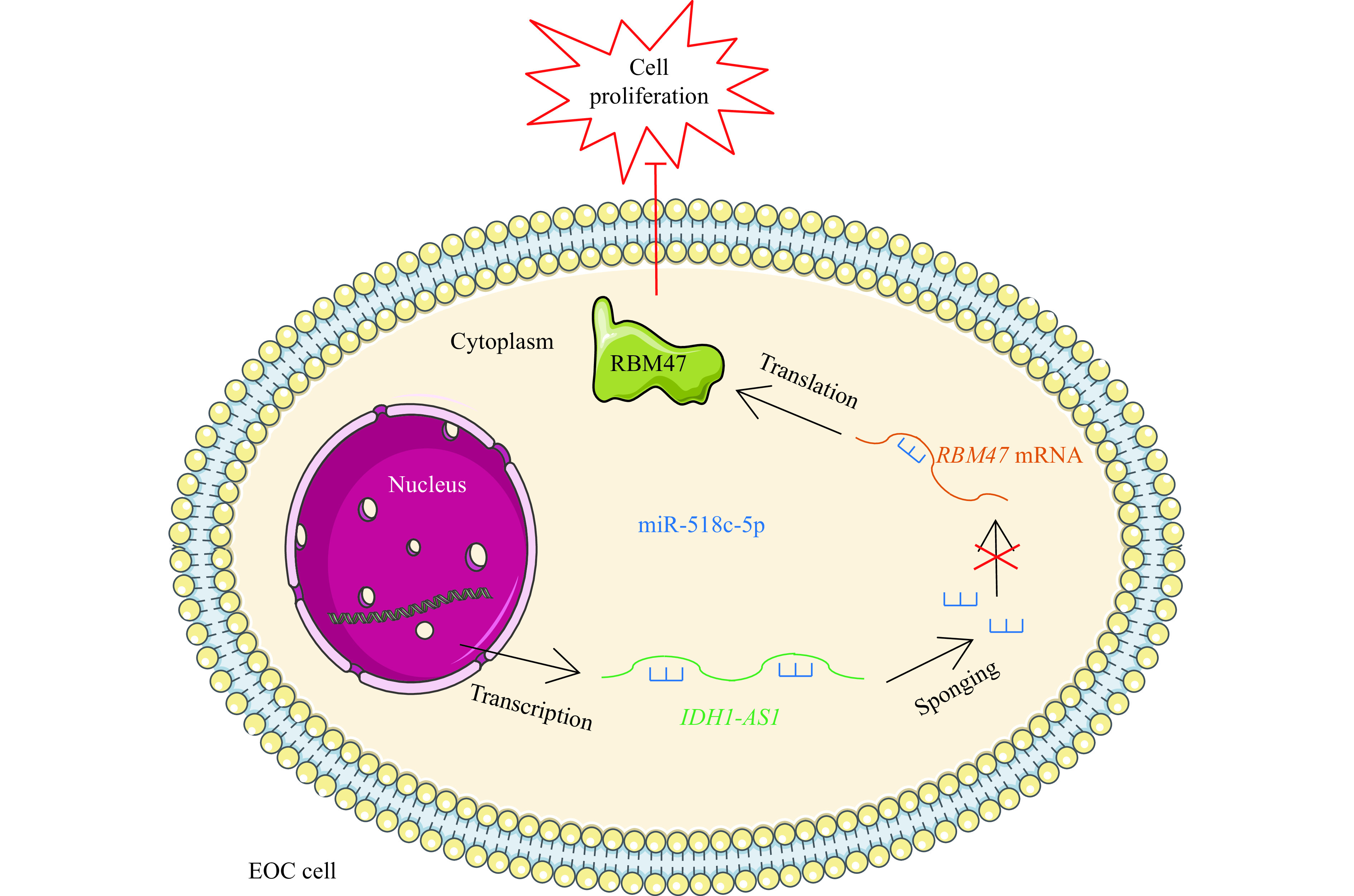
A mechanism schematic of the role of *IDH1-AS1* in EOC.

In conclusion, the present findings reveal that *IDH1-AS1* is downregulated in EOC cells and suppresses EOC cell proliferation and tumor growth. *IDH1-AS1* regulates RBM47 expression levels by a miR-518c-5p-dependent mechanism (***Fig. 6***). Thus, these results indicate that *IDH1-AS1* suppresses cell proliferation in EOC by regulating the miR-518c-5p/RBM47 axis.

However, there were still some deficiencies in the present study. Most EOC patients were in an advanced stage without paracancerous tissues, resulting in a lack of comparison of the *IDH1-AS1* expression in EOC tissues and paracancerous control tissues. In addition, most of the EOC patients from the TCGA database were white, and may not represent the expression of *IDH1-AS1* in Chinese. We have been collecting samples of EOC patients from Chinese, and when the sample size is sufficient, we will conduct clinical data analysis of *IDH1-AS1* in these samples. Taken together, once confirmed by other investigators, the *IDH1-AS1*/miR-518c-5p/RBM47 regulatory axis may be a new treatment target for EOC in the future.

## SUPPLEMENTARY DATA

Supplementary data to this article can be found online.
