# Supplementary material for: LncRNA IDH1-AS1 sponges miR-518c-5p to suppress proliferation of epithelial ovarian cancer cell by targeting RMB47
Source: J Biomed Res. 2023 Nov 20;38(1):51–65. doi: 10.7555/JBR.37.20230097 (PMC10818171; doi:10.7555/JBR.37.20230097)
Supplement: Supplementary file 1 — Supplementary data to this article can be found online. [file jbr-38-51-S1.pdf]

Available online at www.jbr-pub.org.cn

Open Access at PubMed Central

JBR

The Journal of Biomedical Research, 2023 37(0): 1–2

Supplementary Data

LncRNA *IDH1-AS1* sponges miR-518c-5p to suppress proliferation of epithelial ovarian cancer cell by targeting RMB47

Juan Zhou[1,△], Yiran Xu[1,△], Luyao Wang[1], Yu Cong[1], Ke Huang[1], Xinxing Pan[1], Guangquan Liu[1], Wenqu Li[1], Chenchen Dai[1], Pengfei Xu[2,✉], Xuemei Jia[1,✉]

[1]*Department of Gynecology, Women's Hospital of Nanjing Medical University, Nanjing Maternity and Child Health Care Hospital, Nanjing, Jiangsu 210004, China;*

[2]*Nanjing Maternity and Child Health Medical Institute, Women's Hospital of Nanjing Medical University, Nanjing Maternity and Child Health Care Hospital, Nanjing, Jiangsu 210004, China.*

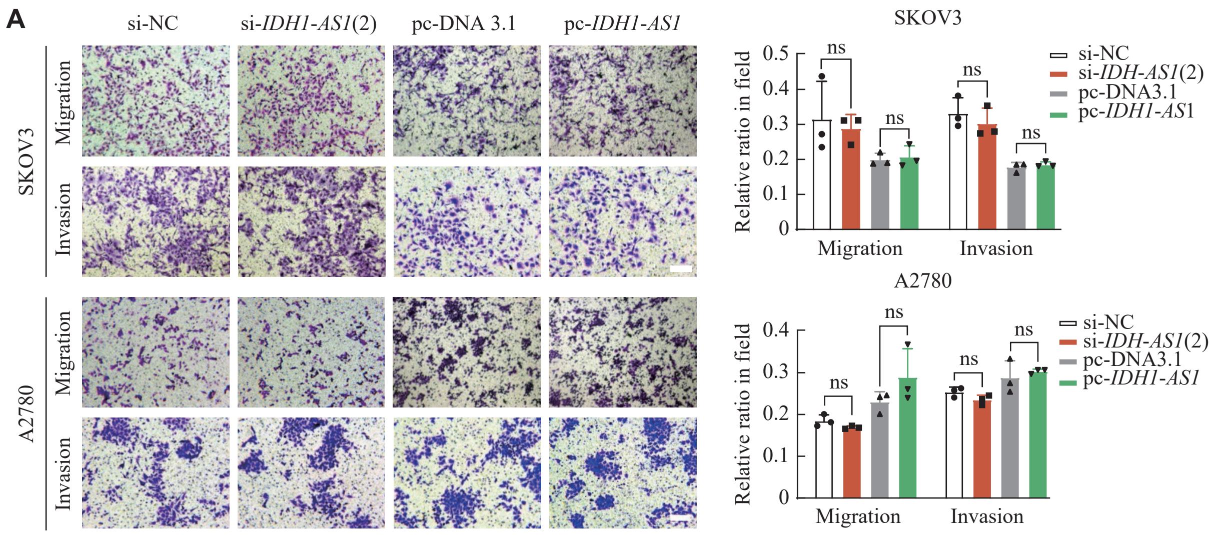

(Continued)

△These authors contributed equally to this work.

✉Corresponding authors: Xuemei Jia, Department of Gynecology, Women's Hospital of Nanjing Medical University, Nanjing Maternity and Child Health Care Hospital, 123 Mochou Rd, Nanjing, Jiangsu 210004, China. Tel: +86-25-52226867, E-mail: xmjia@njmu.edu.cn; Pengfei Xu, Nanjing Maternity and Child Health Medical Institute, Women's Hospital of Nanjing Medical University, Nanjing Maternity and Child Health Care Hospital, 123 Mochou Rd, Nanjing, Jiangsu 210004, China. Tel: +86-25-52226264, E-mail: pengfeixu@njmu.edu.cn.

Received: 18 April 2023; Revised: 22 August 2023; Accepted: 29 August 2023; Published online: 20 November 2023

CLC number: R73.3, Document code: A

The authors reported no conflict of interests.

This is an open access article under the Creative Commons Attribution (CC BY 4.0) license, which permits others to distribute, remix, adapt and build upon this work, for commercial use, provided the original work is properly cited.

© 2023 by the Journal of Biomedical Research.

https://doi.org/10.7555/JBR.37.20230097

(Continued)

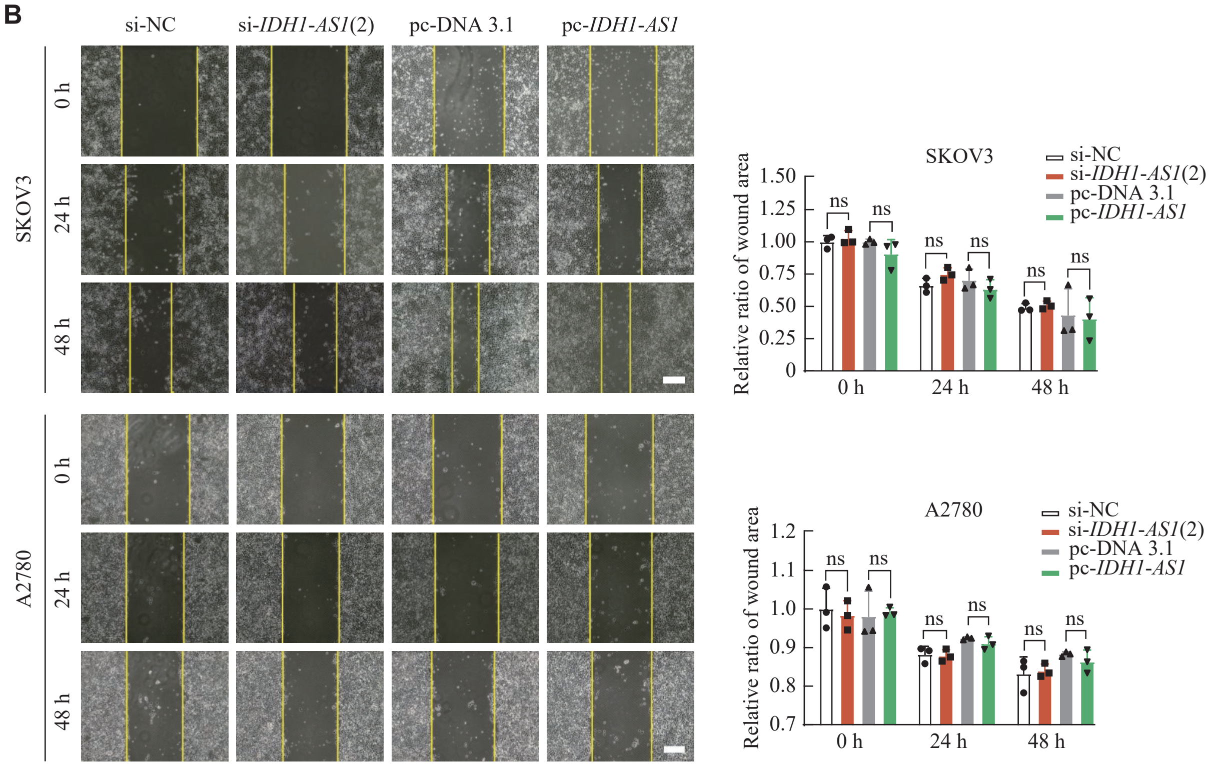

Supplementary Fig. 1 ***IDH1-AS1*** **did not affect the migration and invasion of EOC cells.** A: Migration and invasion of SKOV3 and A2780 cells after transfection of *IDH1-AS1* siRNA and overexpressing plasmid as evaluated by the transwell assay. Scale bar: 200 μm. B: Migration of SKOV3 and A2780 cells after transfection of *IDH1-AS1* siRNA and overexpressing plasmid as evaluated by wound-healing assay. Scale bar: 200 μm. Data were obtained from three independent experiments. Data are presented the mean ± standard deviation. Two-tailed Student's *t*-test was performed to indicate significance. Abbreviations: EOC, epithelial ovarian cancer; siRNA, short interfering RNA; ns, not significant.
